# Endometriosis and the Coronavirus (COVID-19) Pandemic: Clinical Advice and Future Considerations

**DOI:** 10.3389/frph.2020.00005

**Published:** 2020-07-07

**Authors:** Mathew Leonardi, Andrew W. Horne, Mike Armour, Stacey A. Missmer, Horace Roman, Luk Rombauts, Lone Hummelshoj, Arnaud Wattiez, George Condous, Neil P. Johnson

**Affiliations:** ^1^Acute Gynaecology, Early Pregnancy, and Advanced Endoscopy Surgery Unit, Nepean Hospital, Kingswood, NSW, Australia; ^2^Sydney Medical School Nepean, University of Sydney, Sydney, NSW, Australia; ^3^Department of Obstetrics and Gynecology, McMaster University, Hamilton, ON, Canada; ^4^MRC Centre for Reproductive Health, University of Edinburgh, Edinburgh, United Kingdom; ^5^NICM Health Research Institute, Western Sydney University, Penrith, NSW, Australia; ^6^Translational Health Research Institute (THRI), Western Sydney University, Penrith, NSW, Australia; ^7^Department of Epidemiology, Harvard T.H. Chan School of Public Health, Boston, MA, United States; ^8^Boston Center for Endometriosis, Boston Children's Hospital and Brigham & Women's Hospital, Boston, MA, United States; ^9^Department of Obstetrics, Gynecology, and Reproductive Biology College of Human Medicine, Secchia Center, Michigan State University, Grand Rapids, MI, United States; ^10^World Endometriosis Society, Vancouver, BC, Canada; ^11^Endometriosis Centre, Clinic Tivoli-Ducos, Bordeaux, France; ^12^Department of Obstetrics and Gynaecology, Aarhus University Hospital, Aarhus, Denmark; ^13^Department of Obstetrics and Gynaecology, School of Medicine, Monash University, Melbourne, VIC, Australia; ^14^Endometriosis.org, London, United Kingdom; ^15^Latifa Hospital, Dubai, United Arab Emirates; ^16^Department of Obstetrics and Gynecology, University of Strasbourg, Strasbourg, France; ^17^Robinson Research Institute, University of Adelaide, Adelaide, SA, Australia; ^18^Department of Obstetrics and Gynaecology, University of Auckland, Auckland, New Zealand; ^19^Auckland Gynaecology Group and Repromed Auckland, Auckland, New Zealand

**Keywords:** endometriosis, pelvic pain, infertility, COVID-19, coronavirus, recommendations, laparoscopic surgery, assisted reproductive technology

## Introduction

The COVID-19 pandemic has led to a dramatic shift in the clinical practice of women's health and routine care for endometriosis has been severely disrupted. Endometriosis is defined as an inflammatory disease characterized by lesions of endometrial-like tissue outside the uterus that is associated with pelvic pain and/or infertility (1). It affects ~10% of reproductive age women worldwide, is diagnosed by surgical visualization or by radiological imaging, and is managed with hormone treatments or by laparoscopic removal of lesions (2–4).

At the time of writing, under the guidance of international gynecological organizations (5–7), many centers temporarily ceased offering outpatient appointments, diagnostic imaging for non-acute pelvic pain, surgery for endometriosis, and fertility treatments. In the absence of routine care pathways and uncertainty about when health services will be available again, endometriosis sufferers are likely to feel vulnerable and that resultant stress and anxiety may contribute to a worsening of symptoms. The pandemic poses several important questions for healthcare providers on how best to deliver care within these restrictions. Herein, we present clinical advice on the management of endometriosis during the COVID-19 pandemic and future considerations (Table 1).

**Table 1:** Advice summary for endometriosis care during the COVID-19 pandemic and future considerations.

**Be aware of the risks of the COVID-19 pandemic on endometriosis patients**
Reduction in quality of life secondary to ° Delayed diagnosis and treatment due to ▪ the closure of outpatient clinical services (consultations, diagnostic imaging, allied health appointments) and ▪ the eventual increase in the waitlist for services once they resume. °The high degree of uncertainty of surgical or fertility interventions Treatment options for patients with endometriosis
Postponement of elective surgery and fertility therapy should be guided by medical colleges and societies and made by governing bodies Continue current management if symptoms are stable or contact a healthcare provider for changes to medication if symptoms are not well-managed Patients should seek telehealth appointments over in-person visits Patients with pain due to endometriosis may still consider the use of NSAIDs or other over-the-counter analgesic medications Empirical medical therapy with hormonal medications is appropriate in the absence of an imaging or surgical diagnosis Patients should consider the numerous complementary and alternative pain management strategies available via telehealth services Use of the emergency department
Patients should use telehealth services as much as possible before resorting to visiting the emergency department Future considerations for endometriosis management
Resumption of surgery and fertility therapy should be guided by medical colleges and societies and made by governing bodies When surgery resumes, serious consideration should be given to: ° Screening for COVID-19 pre-operatively ° Adopting appropriate PPE behaviors ° Mitigating release of aerosolized gas by modifying surgical techniques Telehealth services should be considered as a viable method of assessment once routine outpatient services resume Self-management strategies should continue to be highly encouraged as adjuncts to traditional management Preoperative triaging tools including advanced clinical algorithms and imaging strategies should be implemented to avoid diagnostic laparoscopy and multiple/repeated surgical procedures.

## Are Endometriosis Patients a High-Risk Population of Becoming Infected With Sars-CoV-2 or Developing More Severe Covid-19 Disease Symptoms?

To date, there is no evidence that those with endometriosis are at increased risk of becoming infected with SARS-CoV-2 or developing COVID-19 disease^1^. A rare subgroup of those with endometriosis have thoracic endometriosis (lesions within the pleural cavity or on the diaphragm). The exact prevalence is unknown but some case series suggest that up to 12% of those with endometriosis have extra-pelvic endometriosis, with the thorax being the most common site (8). In general, there is a paucity of literature labeling this form of endometriosis as a risk factor for respiratory or systemic illness beyond catamenial pneumothorax (9). As such, it is challenging to know whether this group is at increased risk of becoming infected with SARS-CoV-2 or developing COVID-19. Similarly, there is no evidence that COVID-19 will hasten the progression/development of endometriosis. Nonetheless, the pandemic will likely contribute to a reduction in quality of life secondary to a delay in diagnosis and/or the management of endometriosis owing to the temporary closure of outpatient services, (including complementary therapies), postponement of planned surgical or fertility treatments, and an eventual increase in the waitlist for services once they resume. The extent of the impact will depend on the duration of service postponement and regional resource variations (e.g., access to operating theater time when surgeries resume).

## Management Options Available During the Covid-19 Pandemic

We encourage individuals in need of help to seek a clinical assessment with their general practitioners (GP), gynecologists, physiotherapists, and/or complementary medical providers through telehealth avenues or in-person when services resume exercising caution to follow local risk-reduction practices. Referral to a gynecologist with expertise in endometriosis may also be appropriate to offset the new diagnostic and therapeutic challenges faced during this time. Those with an established diagnosis who are seeking help, regardless of their intentions to pursue surgical management, should discuss with their clinician the potential to modify their current medication regimen. Some with suspected endometriosis may accept a clinical diagnosis in the absence of imaging or laparoscopy and empirical medical therapy can be initiated (2). In those given a clinical diagnosis, and who don't respond to medical therapy, non-invasive imaging could be the first investigation arranged when it is safe to do so to evaluate for features that can reliably be identified such as deep endometriosis, ovarian endometriomas, and pouch of Douglas obliteration; whilst recognizing that at present superficial peritoneal endometriosis is not reliably detected using imaging (10, 11). Non-endometriosis pathologies may also be diagnosed. Knowledge of these entities has the potential to change clinical management, so awareness of them is important. However, if a patient is responding well to empiric treatment and does not intend to alter management, it may be reasonable to proceed without imaging. Laparoscopy as a diagnostic tool should be avoided unless the intention is to simultaneously surgically treat any endometriosis that is found (12). This could be considered in those who are experiencing failed medical management, have endometriosis-related infertility seeking to avoid or unable to access assisted reproductive technologies, or simply prefer to undergo surgery instead of using medical management.

Initially, caution in the use of non-steroidal anti-inflammatory drugs (NSAIDs), commonly used for endometriosis-related pain, was being advised (13). At present, the World Health Organization states that there is no evidence of severe adverse events, acute health care utilization, decreased long-term survival, or diminished quality of life in patients with COVID-19, as a result of the use of NSAIDs (14). As such, those with endometriosis-related pain who use NSAIDs can continue to do so as needed, ensuring appropriate dosing according to medication labels and/or healthcare providers, bearing in mind that long-term use of NSAIDs come with its own set of side-effects including peptic ulceration and adverse impact on ovulation (2). Beyond traditional medical therapies, problem-focused interventions such as education, modifying work/school/social life, taking advantage of virtual and telephone support provided by national endometriosis organizations, improving sleep hygiene, low-intensity physical activity (including pelvic exercises, yoga), dietary changes, application of heat, and medical cannabis should be considered, either with the assistance of a healthcare provider via telehealth or independently by patients themselves (15). Similarly, emotion-focused strategies, which include relaxation/mindfulness, acceptance of chronic illness (e.g., via Acceptance and Commitment Therapy with the help of a clinical psychologist through telehealth), reducing catastrophizing, and improving a balance toward positive attitude can be considered (15). These strategies are not unique to the COVID-19 pandemic and are recognized as an integral part of the usual multidisciplinary management of endometriosis.

Patients should be aware that, if they experience acute exacerbations of their chronic pain, they may warrant urgent medical assessment, as such cases, especially those with suspected endometrioma or severe acute recalcitrant exacerbation of pain, may require urgent surgery. However, most pain exacerbations are not life- or organ-threatening and with appropriate counseling and support, a face-to-face consultation in the emergency department may be avoided. Some GPs may find it challenging to confidently reassure patients that they are safe to avoid an emergency department visit, so urgent telehealth consultation with a gynecologist or pain specialist may be helpful. That said, we do not advocate for the avoidance of the emergency department out of fear, so patients and providers should continue to judiciously and safely use this service when warranted.

## Advice On Resuming Pre-Pandemic “Regular” Care for Endometriosis

As restrictions begin to lift, healthcare services, including surgery for endometriosis, will resume. The decision about *when* clinical care should resume will be determined by medical colleges and societies, in compliance with governing bodies informed by emerging viral disease pandemic experts. The provision of appropriate medical and surgical care should resume with an emphasis on safety for patients, healthcare staff, and society. The American College of Surgeons (16), the Royal College of Obstetricians and Gynaecologists (17), and a collaborative effort by nine women's health care societies (18) outline important guidance for resuming surgical practice and reintroducing these procedures. Though endometriosis is a non-malignant disease, we believe it must be treated with high priority due to the major impact it has on quality of life (19). That said, facilities should employ a prioritization policy committee, including a gynecologist with expertise in managing the various facets of endometriosis (surgery, pain management, fertility treatment), to ensure an appropriate strategy is developed across all specialties. Amongst several strategies (16), previously canceled and postponed endometriosis surgeries should be prioritized. An objective priority scoring tool could also be implemented [e.g., MeNTS instrument (20)]. Based on the procedure, disease type, and patient factors that go into this scoring tool, endometriosis surgery would be relatively low risk. Objectively judging the impact of a 2- or 6-week delay on disease outcome is challenging as timing surgical management (e.g., immediate vs. delayed) has never been evaluated (21). It is unlikely for there to be a change in the surgical difficulty/risk with a 2- or 6-week delay (22). For urgent/emergency surgeries that have continued through the COVID-19 pandemic, there has been discussion about the safety of surgery based on theoretical evidence that aerosolization of the virus can occur with ultrasonic/electrosurgery (23). During this time, a minimally invasive surgical approach is being recommended (24) and felt to be lower risk (20). This COVID-19 specific recommendation aligns with the typical approach to endometriosis preceding the pandemic. Benefits include improved visibility of subtle endometriosis lesions (and therefore targeted treatment), decreased blood loss, reduced post-operative pain levels, and shorter in-hospital stay post-operatively. We support the joint statement by several gynecologic surgical societies, where expert opinion recommendations on intraoperative precautions have been put forward (25).

Adequate preoperative screening and diagnosis of SARS-CoV-2 will be an important consideration for the resumption of endometriosis surgery (26). Though most patients undergoing surgery for endometriosis are relatively young and healthy, we must be cognizant of the increased risk in those with perioperative SARS-CoV-2 infection. It has recently been noted that post-operative pulmonary complications occur in half of the patients with perioperative SARS-CoV-2 infection and are associated with high mortality (27).

At this time, we do not believe that the COVID-19 pandemic warrants a sustained change in the overall medical approach to the management of endometriosis (e.g., avoid surgery and favor medical management). Regardless of a pandemic, we encourage healthcare providers to comprehensively counsel patients on the therapeutic options available for each individual with endometriosis. The possible risks and realistic scheduling obstacles secondary to COVID-19 must be part of this conversation, but patients should still retain their autonomy to choose the option that is best for them.

## Future Considerations

We believe that the COVID-19 pandemic can lead to sustained improvements in the care for those with endometriosis. Firstly, there may be an ongoing openness to telehealth (28). This could dramatically minimize the geographic barriers to care that many women experience, and facilitate the development of endometriosis networks of expertise, which is recommended by the World Endometriosis Society (2). Telehealth may also be an appropriate alternative for patients with pain that limits their ability to travel to their healthcare provider in some settings. Secondly, there may be increased awareness to self-management strategies that have always existed, yet were under-utilized (e.g., mindfulness, physical exercise, and diet) until COVID-19 resulted in them becoming valuable tools for patients (15). Finally, the current situation mandates a more discerning approach to surgery now and in the future, so that we “operate sparingly and operate well.” This approach can be guided by preoperative triaging tools including advanced clinical algorithms and imaging strategies (29) to avoid multiple repeated surgical procedures.

## Author's Note

This manuscript has been released as a pre-print in Authorea (30).

## Author Contributions

All authors meet justification criteria of authorship as per ICMJE: substantial contributions to conception and design or acquisition of data or analysis and interpretation of data, drafting the article or revising it critically for important intellectual content, final approval of the version to be published, and agreement to be accountable for all aspects of the work in ensuring that questions related to the accuracy or integrity of any part of the work are appropriately investigated and resolved.

## Conflict of Interest

ML reports grants from Australian Women and Children's Research Foundation, outside the submitted work. AH reports grants from Chief Scientist Office, NIHR EME, MRC, Wellbeing of Women, Ferring, and Roche Diagnostics during the conduct of the study; and honoraria for consultancy for Ferring, Roche, and AbbVie, outside the submitted work. MA reports grants from Metagenics and Spectrum outside the submitted work. SM reports a grant and consulting fees from Abbvie, and consulting fees from Roche outside the submitted work. LR reports personal fees from Monash IVF Group, grants from Ferring Australia, personal fees from Ferring Australia, non-financial support from Merck Serono, non-financial support from MSD, non-financial support from Guerbet, outside the submitted work; and Minority shareholder and Group Medical Director for Monash IVF Group and the President-Elect of the World Endometriosis Society. HR reports personal fees from Olympus, personal fees from Ethicon, personal fees from Nordic Pharma, personal fees from Plasma Surgical Ltd., outside the submitted work. LH reports personal fees from AbbVie, is the chief executive of the World Endometriosis Society, and the owner of Endometriosis.org., outside the submitted work. GC reports personal fees from Roche, personal fees from GE Healthcare, grants from Australian Women and Children's Research Foundation, outside the submitted work. NJ reports personal fees from Guerbet, personal fees from Vifor Pharma, grants and personal fees from Myovant Sciences, grants from AbbVie, personal fees from Roche, outside the submitted work. The remaining author declares that the research was conducted in the absence of any commercial or financial relationships that could be construed as a potential conflict of interest.

## Footnotes

^1^*People Who Are at Higher Risk for Severe Illness | Coronavirus | COVID-19 | CDC*. Available online at: https://www.cdc.gov/coronavirus/2019-ncov/need-extra-precautions/groups-at-higher-risk.html (accessed April 18, 2020).
